# Geographical variation of Crohn's disease residual incidence in the Province of Quebec, Canada

**DOI:** 10.1186/1476-072X-9-22

**Published:** 2010-05-12

**Authors:** Pascal Michel, Laurie St-Onge, Anne-Marie Lowe, Michel Bigras-Poulin, Paul Brassard

**Affiliations:** 1 Laboratory for Foodborne Zoonoses, Public Health Agency of Canada, Saint-Hyacinthe, Quebec, Canada; 2 Groupe de recherche en épidémiologie des zoonoses et santé publique (GREZOSP), Faculty of Veterinary Medicine, Université de Montréal, Saint-Hyacinthe, Quebec, Canada; 3 Faculty of Medicine, Université de Montréal, Montreal, Quebec, Canada; 4 Faculty of Medicine, McGill University, Division of Clinical Epidemiology, McGill University Health Center (MUHC), Montreal, Quebec, Canada

## Abstract

**Background:**

Crohn's disease (CD) is clinically expressed as a chronic affection of the gastrointestinal tract currently known to have a multifactorial etiology involving a complex pathophysiological host response modulated by genetic susceptibilities, demographic determinants and environmental factors. With more than 20 cases per 100,000 person-years, the province of Quebec, Canada is among regions of the world with highest reported occurrence of CD in relation to other places where comparable estimates are available. This ecological study was designed to provide a medium-scale spatial exploration of CD incidence after accounting for the influence of known population and regional determinants. Health records of consulting patients in southern Quebec were compiled from 1995 to 2000 and used to estimate age and sex standardized rates per health area (n = 156). Various statistical models taking into account the regional effect of Jewish ethnicity, aboriginal ancestry, material deprivation, prescription for oral contraceptives, reportable enteric infection incidence, smoking as well as latitude and longitude locations were fitted.

**Results:**

The final regression model presented a coefficient of determination of 22.8% and there was evidence of an eastern trend in the residual incidence (p = 0.018). Overall, the smoothed residual incidence presented a heterogeneous spatial pattern with evidence of patches (multiple health areas) of high, low and contrasting values. Health areas with most extreme incidence residuals where also distributed over the whole province including one area in the metropolitan area of Montreal and others in surrounding areas.

**Conclusions:**

These findings suggest that known populational and regional factors derived through census information only explain a limited fraction of the geographical variation of CD incidence and lead to speculate that the effects of these factors may be incompletely captured (imperfect construction of proxy variables) or that other important factors remain unmeasured. In this view, markers of genetic profiles of homogeneous sub-populations, and other factors linked to agroenvironmental microbial exposure should be further investigated. Once accounting for known factors, it would also be worth comparing adjacent geographical areas demonstrating abrupt changes in residual incidence rates to further explore effect linked to regional factors from those resulting from various reporting systems.

## Background

Ulcerative colitis and Crohn's disease (CD) are the two major types of inflammatory bowel diseases (IBD). CD affects the gastrointestinal tract, from the mouth to the anus and is known to have a multifactorial etiology involving genetic predispositions, demographic determinants and environmental factors [1]. The increasing frequency of IBD has been put in parallel with the development and modernization of the society, but they can also be linked with changes in individuals and life habits [2]. With an estimated 13.4 new cases/10^5 ^persons, Canada has one of the highest rates of Crohn's disease recorded in the world and its epidemiology shows a geographical variation of the incidence distribution [3]. Spatially heterogeneous distributions of the disease have been observed in distinct parts of the world [4,5] and various regional-level risk factors relating to population characteristics and possible environmental exposures have been proposed in explaining the spatial variation in the occurrence of CD [6-10]. A recent study aiming at describing the occurrence and key regional factors linked to the condition in the province of Quebec, Canada, showed that some regional factors, namely the enteric disease incidence, the proportion of people of Jewish ethnicity and the proportion of immigrant people were significant predictors of incidence of CD in this population [11].

Given the current state of knowledge on CD spatial epidemiology, we hypothesized that known regional factors only account for a small fraction of the total geographical variation of CD incidence and that a spatial investigation of residual variation could further revealed geographical patterns that might contribute to our knowledge of the role of environmental or population factors linked to this condition. This analysis is therefore aimed to explicitly describe medium-scale spatial distribution of CD residual incidence adjusted for a set of known regional predictors in the province of Quebec, Canada.

## Methods

### Study setting

This ecological study was designed as a spatial exploration in follow-up to an epidemiological investigation by Lowe *et al. *[11] presenting the association of regional-level factors with CD in the province of Quebec. The geographical unit of interest was defined based on the smallest health division in this province and are referred herein as "health area" (N = 166, according to the 2006 boundaries). Patient records in the provincial databases specifically include the designation of these health areas. To further increase the specificity of the results, and in consideration of sparse population density and relatively large territories, the ten most northern health areas (north of 50 degree of latitude approximately) of the province (regrouping 0.62% of the total provincial population) were excluded from the analysis. The selection of the geographical unit and area of interest were designed in consideration of the stability of the epidemiological measures (standardized incidence rates), the potential for geographical misclassification errors of records, the desire to minimize possible ecological biases and the structure of the spatial connectivity required for the analysis given the geography of the province under study. The population of interest consisted of an average of 7.2 million people (1996 census).

### Data

CD is not a reportable disease in the province of Quebec, Canada. Therefore, cases of CD consisted of residents of Quebec that were entered in the computerized administrative health databases of this province ("Régie de l'Assurance Maladie du Québec", RAMQ and MED-Echo). These databases were developed in the context of the universal insurance program and for recording hospital discharge information. The database on medical service claims includes more than 99% of the population [12]. In order to be reimbursed by the program, physicians submit to the RAMQ a claim for each medical visit, recording the data and location of the medical service as well as the procedure code for the service provided and the ICD-9 code for the diagnosis. Because of its population-based coverage and routine audits, information from these databases is deemed valid and consistent [13,14]. Health records for the period extending from January 1^st^, 1993 to December 31^st^, 2002 were examined and those between 1995 and 2000 were considered for defining incident cases. Details relating to the case definition and validation have been previously described by Bernstein *et al. *[3] and Lowe *et al. *[11]. Cumulative rates (incidence) were calculated using the mid-year 1996 Quebec population for each health area and age-sex direct internal standardization was performed using the overall provincial age-sex distribution.

### Statistical analyses

The statistical modeling strategy aimed primarily at describing the spatial distribution of residual incidence accounting for known population and demographic risk factors. As such, we were not seeking to study other factors than those presented by Lowe *et al*. (2009) [11]. These were: proportions of the population reporting to be immigrants, of Jewish ethnicity and of aboriginal ancestry, as well as measures of material deprivation, number of prescription for oral contraceptives, reportable enteric infection incidence and smoking. These "population-driven" variables were offered and selected in a stepwise approach leading to a reduced model. To test the residual effect of urban/rural location and if geographical trend was left in the reduced model, the latitude and longitude of the ecoumen of the health areas and a variable describing the level of urbanicity of health areas were also tested for significance. Various model structures, namely Poisson, negative-binomial, spatially explicit (spatial error and spatial lag) and linear multivariable models were developed and assessed as for their fit, and interpretation. A multivariate linear model was kept as the main model in consideration of a lack of spatial error or spatial lag effects, and to retain an easily interpretable outcome variable. The outcome variable was designated as the area level cumulative incidence directly standardized for age and sex (DSR). Significance of predictors was derived using a robust variance estimation (Huber and White) accounting for possible cluster effect (broader regional health districts, n = 12). Residual standardized incidence rates were smoothed using the empirical Bayes method to further address the stability of the rate estimates given heterogeneous population sizes. Moran's I statistics was computed for residual incidence rates. Local Indicators of Spatial Autocorrelation (LISA) were also calculated to study possible local clustering pattern. In this analysis, the LISA were represented by local Moran's I values. Health areas with significant LISA were classified as "High-High", "Low-Low", "High-Low" or "Low-High" according to the location of their respective standardized value within the four quadrants of an univariate Moran scatter plot [15]. An inverse distance spatial connectivity matrix based on the centroid of the population ecoumen was used for the spatially explicit regression models and for the LISA. The statistical modeling was performed using the software STATA (Stata/SE 8.2, 2005). Empirical Bayes Smoothing (EBS) and LISA statistics were calculated using the software GeoDA (GeoDa, 0.9.5-i5, 2004).

### Mapping

In map representations, classification of the outcomes of interest (EBS-CD incidence, and EBS-CD incidence residuals) was done using a bi-directional standard deviation method using five classes where the "average" class included values within 0.5 standard deviation of the mean, the "low" and "high" classes included values between 0.5 and 1.0 standard deviation from the mean and the "very high" and "very low" classes included values 1.0 standard deviation from the mean. Classification and map representation were done using the software ArcGIS 9.3 (ESRI, 2008).

## Results

### CD incidence

From January 1^st^, 1995 to December 31^st^, 2000, a total of 10,033 CD cases were recorded over the 156 health areas of southern Quebec, which correspond to a yearly mean crude rate of 23.2 per 10^5 ^person-year. For the 156 health areas under study, the age-sex standardized cumulative incidence was estimated at 13.9 cases per 10^4 ^persons (95% CI: 13.3 - 14.5). There were 70 (44.9%) health areas that were classified as having an average incidence.

### Multivariable regression model

Estimated parameters of the main multivariable linear regression model are presented in Table 1. The dependant variable was CD standardized cumulative incidence. In this main model, the proportion of immigrant people and proportion of population of aboriginal ancestry in the Quebec population both had a statistically significant negative effect (protective effect) on the dependant variable, whereas the variable describing the proportion of population of Jewish ethnicity had a significant positive association with CD standardized cumulative incidence. A non-significant negative association was found between the dependant variable and the variable describing an increasing level of material deprivation (MAT) and a non-significant positive association was found with reportable enteric infections incidence (ENT). Both the MAT and ENT were forced into the model which presented a coefficient of determination of 22.8%. When latitude and longitude variables corresponding to the geographical centroid of the population ecoumen of the health areas were added to the main model, a statistically positive association (P = 0.018) was found for the longitude (eastern trend). A variable describing the urban or rural aspect of the health region was also offered to the main model but was non-significant (P = 0.64).

**Table 1:** Regression Model of Directly Standardized Incidence of CD

					*95% Confidence Interval*	
***Variables***	***Coefficients***	***Robust SE***	***T-values***	***P-values***	***Lower***	***Upper***

Immigration	-0.14	0.01	-12.19	<0.01	-0.16	-0.11
Jewish ethnicity	0.23	0.02	11.98	<0.01	0.19	0.27
Aboriginal ancestry	-0.22	0.05	-4.14	<0.01	-0.34	-0.11
Material deprivation	-0.96	0.49	-1.96	0.07	-2.00	0.09
Reported Enteric infection	0.06	0.05	1.09	0.29	-0.05	0.17
Constant	15.87	1.03	15.38	<0.01	13.66	18.08

Regression model coefficients were estimated using robust standard error procedure. Model includes the regional effect of immigration, population of Jewish ethnicity, population of aboriginal ancestry, material deprivation and incidence of enteric diseases (n = 156), R2 = 0.2284.

### Distribution of EBS standardized incidence and residual incidence rates

**Figure 1:**
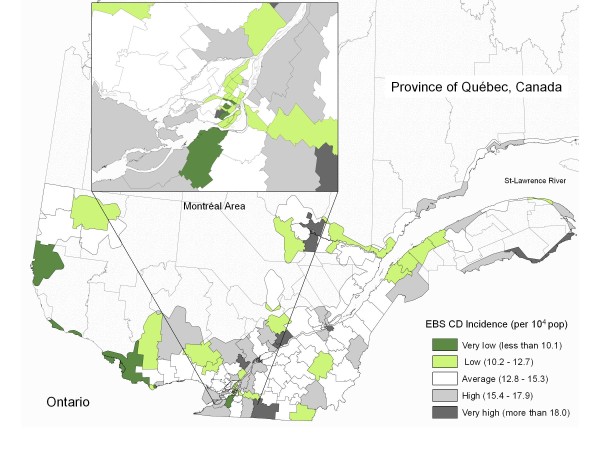
**Crohn's Disease Standardized Cumulative Incidence (1995 - 2000)**. Incidence rates were transformed using empirical Bayesian smoothing function (EBS) and represent age and sex directly standardized cumulative measures. The classification is based on a standard deviation bi-directional method.

**Figure 2:**
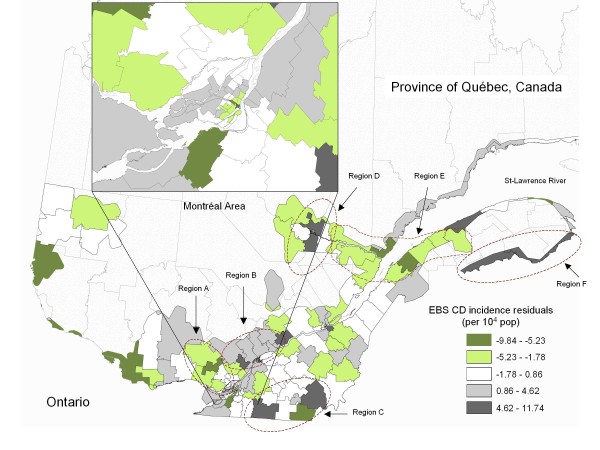
**Crohn's Disease Residual Incidence (1995 - 2000)**. Values represents empirical Bayesian smoothed (EBS) incidence residuals from a regression model adjusting for the regional effect of immigration, population of Jewish ethnicity, population of Aboriginal ancestry, incidence of enteric diseases and socio-economic status. Positive values reflect a larger observed incidence than predicted by the model (under-estimation) and negative values reflect a smaller observed incidence than the one predicted by the model (over-estimation). Patches of areas with lower values (i.e. regions A and E), higher values (i.e. regions B and F) and with discordant values (i.e. regions D and C) are illustrated.

The spatial distribution of the EBS CD standardized cumulative incidence in southern Quebec followed an overall heterogeneous pattern. Some regions included health areas with very high rates, other regions showed homogeneous patches of health areas with low and very low rates and several locations showed a combination of high and low incidence, the highest incidence areas being adjacent to lower incidence areas (Figure 1). After accounting for the effect of selected factors in the main model (Table 1), the spatial distribution of regional EBS cumulative incidence residuals was also heterogeneous, presenting a similar but more contrasted pattern when compared to the distribution of the incidence rates. In Figure 2, positive values reflect a larger observed incidence than the one predicted by the model (under-estimation by the model) and negative values reflect a smaller observed incidence than the one predicted by the model (over-estimation by the model). Patches of health areas with lower values such as regions identified as A and E, higher values (i.e. regions B and F) and with discordant values (i.e. regions D and C) were spread over the province with no specific geographical pattern. For the Island of Montreal (Figure 2, inset), the areas corresponding to the greater downtown sectors had, for the most part, large or very large negative residuals and the rest of the island presented overall large positive residuals. For Quebec city (not shown), the second largest metropolitan area after Montreal in this province, the upper town area (including the business downtown and tourist center) presented large negative residuals and the lower town area, the sub-urban northern and southern areas of the city showed large and very large positive residuals.

### Spatial autocorrelation

**Figure 3:**
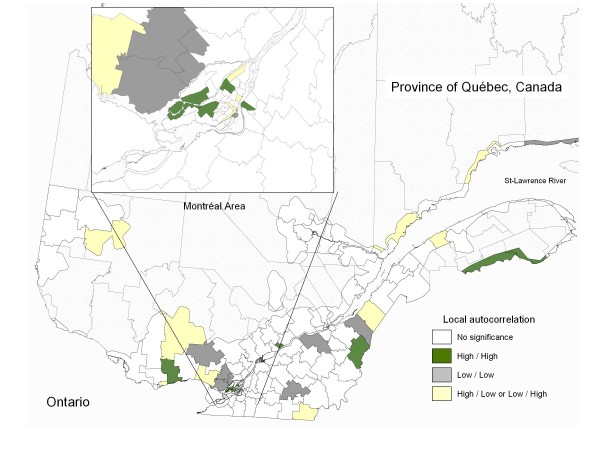
**Local Indicators Of Spatial Autocorrelation (LISA) Of CD Residual Incidence**. Significant negative spatial association (Low/Low) is shown in grey while significant positive association is colored green (High/High). Areas that had non-significant LISA values are represented with no color shading.

There was evidence of statistically significant (P < 0.05) local pockets of spatial autocorrelation (local area of clustering), both for positive values and negative values for the residual incidence of CD in southern Quebec (Figure 3). A total of 11 areas had significant LISA for positive residuals (high/high). Among these, we noted that six of them were in the greater Montreal area. A total of nine areas had significant LISA for negative residuals (low/low) with four of these clustering in a large area north of Montreal (Figure 3 - inset) and the others in various locations. There were also 14 areas with significant LISA values and describing discordance between areas with positive and negative values (high/low or low/high). The overall spatial distribution of these local indices led to no evidence of global spatial autocorrelation (Moran's I = -0.000263, P = 0.30).

### Most extreme values

**Figure 4:**
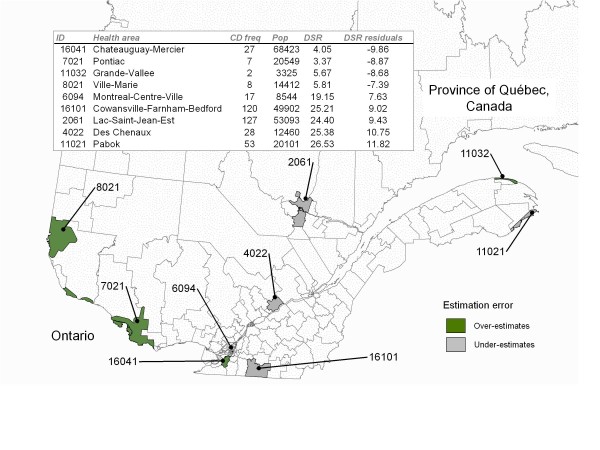
**Health Areas With Most Extreme Incidence Residuals Of CD (1995 - 2000)**. Health areas in Quebec with largest residuals according to a regression model accounting for known regional risk factors. Residual values were smoothed using an empirical Bayes (EBS) method. EBS-residuals larger and smaller than two standard deviations from the mean (zero) were tagged as "largest residuals". Areas in green reflect locations of largest residuals corresponding to an overestimation of the statistical model and areas in grey reveal locations of largest residuals corresponding to an underestimation of the model.

A total of nine health areas in Quebec were described as "areas with largest residuals" after accounting for the expected effect of the known regional risk factors (Figure 4). Of these, five had large positive EBS residual values and four had large negative EBS residuals values. Both positive and negative largest residual values areas were located in various location of the study area.

## Discussion

Although Crohn's Disease (CD) has been recognized as an important health condition in various developed countries for many years, the understanding of its epidemiology is still incomplete [1]. One of the important goals in studying the geographical distribution of complex health conditions such as CD lies on the desire to identify consistent, specific and influential exposures as well as determinants on which medical and public health actions could be targeted. This retrospective investigation of Quebec CD data allowed us to emphasize an apparent spatial heterogeneity observed in the distribution of the CD incidence residuals once controlling for effects from various known factors. Local aggregations (clustering) of areas with high, low or mixed (high/low) residual incidence rates were noted in various locations of the province with no clear pattern.

As discussed by Elliot *et al.*, the observation of an apparent spatially heterogeneous distribution of a health condition may reflect the overall variations of multiple interconnected factors that might play a role of variable importance in the etiology of that condition [16]. Factors such as smoking habits, socio-economical status, urban setting, immigration and specific ethnic profiles such as Ashkenazi Jewish and Aboriginal origins are among recently reported factors that have been highlighted as modulating the regional occurrence of the condition. The possible contribution of these broad factors have led to various theories and speculations implicating the possible effect of diet [17-20], occupation [21-23], allergens [24,25], sunlight [26,27] and microbial exposure in the risk of developing CD [28,29]. In the context of a multifactorial disease such as CD, the heterogeneous spatial distribution might reflect therefore the complex interaction of many factors, each of them with a specific local relationship with population and environmental characteristics. Further characterization of these influences in term of their small and medium scale geographical pattern, and their assessment as to improving geographical models for CD incidence could be of great value in the effort of decomposing the apparent complex geographical variation of this condition.

In this study, an attempt was made to remove effects from a few known regional factors and the analysis of residual values of CD incidence revealed a distribution somehow similar to, but more contrasted than the one describing the non-adjusted CD incidence. This result suggests that important variables might be missing or that the construction of those selected may be imperfect. Using various modeling strategies, our data consistently suggested that approximately 20% of the overall regional variation in CD incidence in the province was explained by the combined effect of immigration, Jewish origin, Aboriginal origin, the level of enteric infection and a measure of socio-economic status. These observations are consistent with what has been previously shown in another Canadian province [30]. However, the construction of variables such as "proportion of immigrant" and "proportion of population from a Jewish ethnicity" from census information are likely to represent incomplete proxy measures for capturing the potential effect of sub-population genetic profiles that could be linked to CD. At a smaller scale, the contrasted pattern seen both in Montreal and Quebec cities may also suggest that other important social factors are still missing in such analyses to explain differences in residual incidence.

As CD is known to be modulated by specific genetic markers [31], the possible importance of genetic susceptibility of homogeneous patches of sub-populations is another important element when interpreting the data from the province of Quebec. The unique population genetic structure of this province is well documented and population characteristics consequent to founder effect, genetic drift and endogamy are known to have influenced the current prevalence and regional distribution of several genetic diseases [32-36]. As proposed by Laberge *et al. *[32], these characteristics have consequences not only on the distribution of monogenic diseases but also on other health conditions modulated by genetic susceptibility. Further attention should be given to investigate the possible link between the genetic profile of sub-populations of this province and regional clusters of high or low CD incidence residuals as illustrated in figure 2 and 4 of this study.

We also described a significant west to east increasing trend in the incidence residuals for the province of Quebec, which corresponds to the trend observed by Bernstein *et al*. [37] for the rest of Canada. Given the heterogeneous pattern in the spatial distribution of incidence residuals, the location of health areas with most extreme values and the relative small territory under study (one province), the eastern trend in the incidence residuals has to be interpreted with caution. It might only reflect that fewer health areas with large negative residuals were along the western border of the province and the areas with the largest positive residual were located at the very eastern side of the province. This finding, however, raises the interest of investigating carefully the CD incidence rates in regions adjacent to these "extreme" values, along the eastern border of the Ontario province, along the northern areas of the states of Vermont and New York, and across the Atlantic provinces of Canada. Under the assumption that environmental physical factors have common influence over both side of the administrative boundaries, these cross-border assessment of CD occurrence might provide initial clues to discriminate possible effect of health systems (surveillance and diagnostic efforts) from environmental or population effects for these regions. Of course, this west-to-east apparent trend, both at the national and provincial level is intriguing and may also reveals true but unmeasured effects linked to the genetic profile of regional populations, cultural factors (diet, traditions) or broader factors influencing environmental conditions such as the climate, soil composition or agricultural practices.

It is also worth noting that our data revealed that the CD incidence residuals were not influenced by the rural/urban profile of the health regions in the southern part of the province of Quebec. Several studies have reported a regional association between CD occurrence and urbanicity, some describing a positive association with urban location [37-40], some describing a positive association with rural location [41] and others not finding any significant associations [30,42,43]. From this, we may suggest that this proxy variable may capture a mixture of unmeasured but geographically heterogeneous or dynamic environmental exposures including effects from microbial quality of water, contact with animals, or occupation which should be further investigated and incorporated in geographical models.

Several other communications have proposed sets of environmental variables comparable to the one used in this analysis and other groups have studied the association between CD and water supply [44], early-life exposures with antibiotic use [45], food and intestinal microbes [46] and have shown some significant associations, but very few details were given on any aspects of the model fit and performance. If we put aside considerations relative to model specifications (i.e. whether we use a negative-binomial model or an explicit spatial model) and the inherent effect of ecological bias affecting inevitably a design such as the one used in this study, the most common reasons affecting a model with relative low performance often include: 1. the lack of important variables; 2. the inadequate or unspecific construction of one or many variables; 3. inadequate choice of the geographical unit in relation to the distribution of the predictors; and 4. data errors and misclassification. In a geographical study like ours, misclassification bias may arise mainly from two components, namely from the application of the outcome definition (classifying a patient as CD) and from geo-positioning cases within a given health area. We have no reason to believe that the misclassification, for both of these components, would be differential. In this case, the consequence of this bias often leads to conservative estimates from the model [47]. The RAMQ database has also been used to study other health conditions and found to provide a valid description of outcomes [48].

Due to the exploratory nature of the current analysis, it was not possible to fully assess the relative contribution of these factors on the performance of our model. However, given the marked effort that was given in this study to minimize possible biases that might be attributable to the construction of variables, misclassification, model specification and geographical unit, we postulate that important regional-level factors are still missing as key predictors to the geographical variation of CD incidence rates. The above considerations, namely the spatial heterogeneity and the likelihood of missing important environmental factors represent further evidence in support of a complex interaction of population-level factors and triggers from the physical and social environment in explaining CD epidemiology.

## Conclusions

Our study suggest that the geographical distribution of incident cases of Crohn's disease in the province of Quebec presented a marked spatial heterogeneity above the one that could be explained by the distribution of regional factors used in this analysis. This finding further supports the presence of unmeasured regional components to the etiology of CD, potentially acting as a trigger or as a necessary co-factor to the development of the disease. CD is a complex disease and other social, genetic, and environmental factors potentially characterizing geographical areas with incidence rates well above or below average should be further investigated.

## Competing interests

The authors declare that they have no competing interests.

## Authors' contributions

Pascal Michel, Laurie St-Onge, Anne-Marie Lowe, Michel Bigras-Poulin, and Paul Brassard were responsible for all aspects of the production of this manuscript including the study design, data extraction, analysis, interpretation of results and discussion. All authors have read and approved the final version of this manuscript.
